# Phosphodiesterase Type 5 Inhibitors Greatly Affect Physicochemical Properties of Model Lipid Membranes

**DOI:** 10.3390/membranes11110893

**Published:** 2021-11-19

**Authors:** Anastasiia A. Zakharova, Svetlana S. Efimova, Olga S. Ostroumova

**Affiliations:** Institute of Cytology of Russian Academy of Sciences, Tikhoretsky 4, 194064 Saint Petersburg, Russia; efimova@incras.ru (S.S.E.); ostroumova@incras.ru (O.S.O.)

**Keywords:** phosphodiesterase type 5 inhibitors, sildenafil, vardenafil, tadalafil, lipid bilayers, liposomes, membrane boundary potential, lipid melting, ion channel, gramicidin A, nystatin

## Abstract

Although phosphodiesterase type 5 inhibitors are widely used and well-studied drugs, the potential benefits of their application in the treatment of various diseases and new drug delivery systems, including liposome forms, are still being discussed. In this regard, the role of the lipid matrix of cell membranes in the pharmacological action of the inhibitors is of special interest. It was shown that sildenafil, vardenafil, and tadalafil caused a significant decrease in the boundary potential of model membranes composed of palmitoyloleoylphosphatidylcholine or its mixture with cholesterol, by 70–80 mV. The reduction in the membrane dipole potential induced by inhibitors led to a 20–25% increase in the conductance of cation-selective pores formed by the antimicrobial peptide gramicidin A. The addition of sildenafil or vardenafil also led to a significant decrease in the temperature of the main phase transition of dipalmytoylphosphatidylcholine, by about 1.5 °C, while tadalafil did not change the melting temperature. Sildenafil, vardenafil, and tadalafil enhanced the pore-forming activity of the antifungal polyene antibiotic nystatin by 11, 13, and 2 times, respectively. This fact might indicate the induction of membrane curvature stress by the inhibitors. The data obtained might be of special interest for the development of lipid-mediated forms of drugs.

## 1. Introduction

For decades, sildenafil, vardenafil, and tadalafil have been used to treat erectile dysfunction through the selective inhibition of cGMP-specific phosphodiesterase type 5 (PDE-5), which is responsible for cGMP degradation in the corpus cavernosum. The vasodilating and protective properties of PDE-5 inhibitors allow these drugs to be used as first-line treatment for a number of serious diseases, including pulmonary and renal hypertension, prostatitis, ischemic lesions of various organs, and in vitro fertilization. In particular, sildenafil has been shown to increase endometrial thickness and to contribute to increasing the chances of getting pregnant for women with various lesions affecting uterine functions [1,2,3,4,5].

At present, sildenafil is administered only by the oral route [6]. Oral administration of the drug is characterized by a significant reduction in the bioavailability and pharmacological activity due to the hepatic first-pass metabolism [7]. According to Nichols et al. [8], the oral bioavailability was found to be only about 40% due to first-pass metabolism. A delayed onset of the pharmacological effect, which usually started within 45 min after dosing, was also reported [9]. Moreover, upon oral administration the onset of action is affected by food, especially fat-rich meals [10]. In addition, treatment with PDE-5 inhibitors requires repeated doses to sustain drug plasma levels and is concomitant with numerous side effects such as blood pressure reduction, headaches, flushing, and nasal congestion [7,8,11].

Delivery of PDE-5 inhibitors through a local tissue area would be considered an alternative to the oral route, in order to avoid systemic adverse side effects, to increase the bioavailability, to shorten the onset time, and to sustain the effect for a longer period. Recently, the application of PDE-5 inhibitors in transdermal (thin films and suspensions) and intravaginal (suppositories and gelling sponges) forms has been reported [2,12,13,14,15].

In many cases, the authors proposed the use of liposomes as a universal delivery system. Liposomes have the distinct advantages of being both nontoxic and biodegradable because they are composed of naturally occurring substances such as phospholipids and cholesterol. Liposomal drug delivery systems also have the ability to carry both hydrophilic and lipophilic drugs and to improve undesirable drug properties such as low solubility and poor membrane permeability [16,17,18]. However, it should be emphasized that the development of liposomal dosage forms of PDE-5 inhibitors requires detailed study of the mechanisms of their interaction with lipids. In particular, the pharmacological properties of liposomal formulations, including drug encapsulation efficiency, greatly depend on vesicle composition. According to Rafai et al. [19], sildenafil loading to liposomes decreased with an increase in cholesterol concentration. Hosny et al. [20] showed that avanafil encapsulation efficiency in dipalmytoylphosphatidylcholine (DPPC)-containing liposomes is characterized by higher values than in egg phosphatidylcholine-containing vesicles. Moreover, avanafil loading nonlinearly depended on the cholesterol content: it increased with an increase in cholesterol concentration to a certain limit, and then, upon reaching this limit, it decreased with further increase in cholesterol content [20]. The authors supposed that avanafil encapsulation depends on the fluidity of the liposome membranes. Furthermore, the drugs themselves might affect the membrane lipid ordering, which reduces liposome stability. Thus, for the development of new dosage forms of PDE-5 inhibitors, it is relevant to study the effects of the compounds on the physicochemical properties of artificial lipid membranes of various compositions.

In this study, we demonstrated the ability of PDE-5 inhibitors to affect the transmembrane distribution of electrostatic potentials and lipid packing. In addition, we estimated the role of changes in the physicochemical properties of the membranes caused by PDE-5 inhibitors in the regulation of the pore-forming activity of an antimicrobial peptide, gramicidin A, and an antifungal polyene macrolide antibiotic, nystatin.

## 2. Materials and Methods

### 2.1. Materials

All chemicals used were of reagent grade. Synthetic 1-palmitoyl-2-oleoyl-*sn*-glycero-3-phosphocholine (POPC), 1-palmitoyl-2-oleoyl-*sn*-glycero-3-phospho-(1′-rac-glycerol) (POPG), 1,2-dioleoyl-*sn*-glycero-3-phosphocholine (DOPC), 1,2-dipalmitoyl-sn-glycero-3- phosphocholine (DPPC), and cholesterol (Chol) were obtained from Avanti^®^ Polar Lipids (Alabaster, Alabama, AL, USA). Nonactin, di-8-ANEPPS, KCl, HEPES, KOH, DMSO, gramicidin A (GrA), nystatin (Nys), sildenafil citrate, vardenafil hydrochloride, and tadalafil were purchased from Sigma-Aldrich^®^ (Merck KGaA, Darmstadt, Germany). The chemical structures of the tested PDE-5 inhibitors are presented in Table 1.

KCl solutions (0.1 M or 2.0 M) were buffered using 5 mM HEPES–KOH at pH 7.4. All experiments were performed at room temperature (25 ± 2 °C).

### 2.2. Membrane Boundary Potential Measurements

Planar lipid bilayers were prepared using a monolayer opposition technique [26]. Lipid membranes were formed from condensed lipid monolayers consisting of pure POPC or mixtures of POPC/Chol (67/33 mol%) and POPC/POPG (50/50 mol%) on a 50 µm diameter aperture in a 10 µm thick Teflon film separating two (*cis*- and *trans*-) compartments of a Teflon chamber. The aperture was pretreated with hexadecane. Solutions of 0.1 M KCl were buffered using 5 mM HEPES–KOH at pH 7.4.

Ag/AgCl electrodes with 1.5% agarose/2 M KCl bridges were used to apply voltage (*V*) and measure transmembrane current. Here, “positive voltage” refers to the case in which the *cis*-side compartment is positive with respect to the *trans*-side. The current was measured using an Axopatch 200B amplifier (Molecular Devices, San Jose, CA, USA) in the voltage clamp mode. Data were digitized using a Digidata 1440A and analyzed using pClamp 10.0 (Molecular Devices, San Jose, CA, USA) and Origin 8.0 (OriginLab Corporation, Northampton, MA, USA). Data were acquired at a sampling frequency of 5 kHz using low-pass filtering at 1 kHz, and the current tracks were processed through an 8-pole Bessel 100 kHz filter.

The steady-state transmembrane current induced by K^+^-nonactin was modulated via the two-side addition of stock solutions of sildenafil and vardenafil in DMSO, and tadalafil in ethanol to the membrane bathing solution to obtain a final concentration ranging from 5 to 240 μM. It was assumed that the membrane conductance (*G*), defined as the ratio of the transmembrane current to the applied voltage (*V* = 50 mV), is related to the membrane boundary potential (*φ_b_*), the potential jump between the membrane interior and aqueous solution bulk, by the Boltzmann distribution [27]. To assess the changes in the membrane boundary potential (Δ*φ_b_*), the ratio of transmembrane current after (*G_IPDE-_*_5_) and before (*G*_0_) addition of PDE-5 inhibitor was determined:

(1) $$\frac{G_{IPDE-5}}{G_{0}}=\exp(-\frac{ze\Delta \phi_{b}}{kT})$$

where *ze* is the ion charge, *k* is the Boltzmann constant, and *T* is the absolute temperature.

The Langmuir adsorption isotherm was used to describe the dependence of the changes in the membrane boundary potential (Δ*φ_b_*(*C*)) on the PDE-5 inhibitor concentration (*C*) [28,29,30,31,32]:

(2) $$\Delta \phi_{b}(C)=\frac{\Delta \phi_{b}(\max)C}{C+K}$$

where Δφb(max) is the maximum changes in the membrane boundary potential at an infinitely high concentration of PDE-5 inhibitor, and K is the desorption constant of the PDE-5 inhibitor, which characterizes an inverse affinity between the inhibitor and the membrane. The slope coefficient of the linear dependence of [Δφb(max)]/[Δφb(C)] on 1/C is equal to K. The linear fitting was performed by Origin 8.0 (OriginLab Corporation, Northampton, MA, USA). The changes in the φb for the defined experimental conditions were averaged from 3 to 7 bilayers (mean ± sd, *p* ≤ 0.05).

### 2.3. Membrane Dipole Potential Measurements

To prepare large unilamellar vesicles containing 1 mol% of di-8-ANEPPS, a mini-extruder (Avanti^®^ Polar Lipids, Alabaster, AL, USA) was used. A stock solution of pure POPC or DOPC in chloroform was mixed with 1 mM ethanol solution of di-8-ANEPPS and twice resuspended in pentane. After desiccation of the lipid solution under a gentle stream of nitrogen, the resulting lipid film was hydrated with a buffer solution (0.1 M KCl, 5 mM HEPES–KOH, pH 7.4). The suspension was subjected to five freeze/thaw cycles and then passed through a polycarbonate membrane with a nucleopore diameter of 100 nm by extrusion 13 times to obtain unilamellar vesicles. After that, PDE-5 inhibitors were added to the liposome suspension to obtain a final concentration in the range from 5 to 240 μM. The steady-state fluorescence measurements were performed with a Fluorat-02-Panorama spectrofluorometer (Lumex, Saint-Petersburg, Russia) at room temperature. The fluorescence excitation ratio *R* was defined as the ratio of the fluorescence intensity at excitation wavelengths of 420 nm and 520 nm and at an emission wavelength of 670 nm to compensate for any influences of membrane fluidity alteration [33]. The changes in the membrane dipole potential (*ϕ_d_*) of POPC and DOPC bilayers were estimated using obtained values of *R* as described by Starke-Peterkovic et al. [34]. The changes in the *ϕ_d_* for the defined experimental conditions were averaged from 3 to 7 bilayers (mean ± sd, *p* ≤ 0.05).

### 2.4. Differential Scanning Microcalorimetry

Giant unilamellar vesicles were prepared from a liposome suspension containing 3 mM of pure DPPC and buffer solution (5 mM HEPES–KOH at pH 7.4) by the electroformation method using Vesicle Prep Pro (Nanion Technologies, Munich, Germany) (standard protocol; 3 V, 10 Hz, 1 h, 55 °C). The tested PDE-5 inhibitors were added to aliquots to obtain final concentrations of 10, 50, and 100 μM. The liposomal suspension was heated at a constant rate of 0.2 °C·min^−1^ using a μDSC 7EVO microcalorimeter (Setaram, Caluire-et-Cuire, France). The reversibility of the thermal transitions was assessed by reheating the sample immediately after the cooling step from the previous scan. The temperature dependence of the excess heat capacity was analyzed using Calisto Processing (Setaram, Caluire-et-Cuire, France). The thermal behavior of the lipids in the absence and presence of sildenafil, vardenafil, and tadalafil was described by the changes in the temperature of the pretransition peak (Δ*T_p_*), the change in the maximum temperature of the main phase transition (Δ*T_m_*), and the change in the half-width of the main peak (Δ*T*_1/2_). The magnitudes of *T_m_* and *T*_1/2_ were averaged from 3 to 4 independent experiments (mean ± sd, *p* ≤ 0.05).

### 2.5. Reconstitution of Ion Channels into Planar Lipid Bilayers

Planar lipid bilayers were made using Montal and Muller’s technique [26] from POPC or POPC/Chol mixture as described above. Lipid bilayers were bathed in 2.0 M KCl at pH 7.4. After the membrane was completely formed and stabilized, stock solutions of GrA (in ethanol) or Nys (in DMSO) were added to the aqueous phase on the *cis*-side or both sides of the bilayer to obtain a final concentration of 6–17 μM and 0.1–0.3 mM, respectively. The tested PDE-5 inhibitors were added to both sides of the membrane up to 100 μM.

The conductance of GrA single channels (*g*) was defined as the ratio between the current flowing through a single GrA pore (*i*) and *V*. The conductance fluctuation histograms were made for the constant transmembrane voltages. The total number of events used for the channel fluctuation analysis was 500–1000. Peaks on the conductance histograms were fitted by the normal density. The χ^2^ criterion was applied to verify the distribution hypothesis (*p* < 0.05).

A steady-state Nys-induced transmembrane current was used to assess the channel-forming activity of polyene macrolide after and before two-sided additions of the tested PDE-5 inhibitors. The mean ratios (*I_∞_*/*I_∞_*^0^) of the steady-state macroscopic currents after (*I_∞_*) and before (*I_∞_*^0^) two-sided modifier addition were averaged from 5 to 7 bilayers (mean ± sd, *p* ≤ 0.05).

## 3. Results and Discussion

According to Park et al. [21] and Gobry et al. [22], the octanol/water partition coefficient of sildenafil is about 2.7 (Table 1). The octanol/water partition coefficient of structurally similar vardenafil is slightly higher and is equal to 3.6 (Table 1), probably due to the replacement of a methyl group in the piperazine ring (of sildenafil) with an ethyl group. Tadalafil is characterized by a different structure compared with structurally related sildenafil and vardenafil and has a *logP*_o/w_ value of about 2.4 [23]. Considering the high lipophilicity of PDE-5 inhibitor molecules, they are thought to interact with membranes and affect their physicochemical properties.

### 3.1. The Effects of PDE-5 Inhibitors on the Electrical Properties of Lipid Bilayers and Voltage-Sensitive Ion Channels

**Figure 1:**
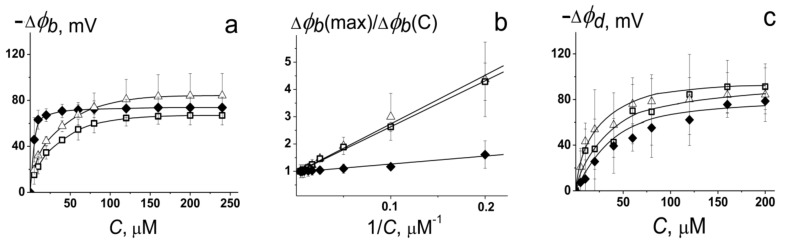
(**a**) The dependence of the changes in the boundary potential of the POPC membranes (Δ*φ_b_*) on the concentration of sildenafil (□), vardenafil (Δ), and tadalafil (♦) in the aqueous solution. The bilayer bathed in 0.1 M KCl at pH 7.4 (*V* = 50 mV). (**b**) The dependences of (Δ*φ_b_*(max)/Δ*φ_b_*(*C*)) on 1/*C* in POPC membranes. (**c**) The dependence of the changes in the dipole potential of the POPC membranes (Δ*φ_d_*) on the concentration of tested PDE-5 inhibitors.

The alterations in the transmembrane distribution of the electrical potentials induced by the PDE-5 inhibitors were estimated by the changes in the steady-state K^+^-nonactin-produced transmembrane current. POPC and cholesterol were used as model zwitterionic lipids, which are abundant in plasma membranes of mammalian cells. Figure 1a shows the dependences of changes in the boundary potential (Δ*φ_b_*) of POPC bilayers on the concentration of PDE-5 inhibitors. One can see that sildenafil, vardenafil, and tadalafil substantially reduce *φ_b_*. Moreover, the boundary potential of bilayers decreases sharply as the solution concentration of the PDE-5 inhibitors increases in the range from 5 to 120 μM. A further increase in the concentration of the tested agents does not lead to changes in *φ_b_*. Previously, Ostroumova et al. [31,32,35,36] showed that the adsorption of plant polyphenols and alkaloids on model lipid membranes is satisfactorily described by the Langmuir adsorption isotherm with the characteristic parameters: the maximum changes in the boundary potential of bilayers at an infinitely high concentration of the agent (−Δ*φ_b_*(max)) and its desorption constant (*K*). The linearity of the dependence of Δ*φ_b_(*max)/Δ*φ_b_*(*C*) on 1/*C* (Figure 1b) demonstrated the applicability of the Langmuir adsorption isotherm to the description of the adsorption of the PDE-5 inhibitors on the phospholipid bilayers.

Table 2 shows the characteristic parameters of the Langmuir adsorption isotherm, Δ*φ_b_*(max) and *K*, for different PDE-5 inhibitors in POPC membranes. Sildenafil, vardenafil, and tadalafil decrease Δ*φ_b_*(max) of POPC membranes by about 70, 80, and 70 mV, respectively. The linear approximation of dependences of Δ*φ_b_*(max)/Δ*φ_b_*(*C*) on 1/*C* shows that *K*-values of sildenafil and vardenafil are about 20 µM, while the same parameter for tadalafil is about 3 µM. The desorption constant characterizes the inverse affinity of the compound to the membrane and might depend on its lipophilicity, charge, and polarity. For this reason, on the basis of the lower values of *logP*_o/w_ and the dipole moment (µ) of tadalafil (Table 1), a higher *K*-value of tadalafil compared with sildenafil and vardenafil should be expected. However, tadalafil is characterized by a lower *K*-value (Table 2) (greater membrane affinity) and demonstrates significant potential-modifying ability at lower concentrations than the other tested PDE-5 inhibitors (Figure 1a). Probably, the key role is played by the orientation of the dipole moment of tadalafil along the normal to the membrane surface, while the projections of the dipole moments of sildenafil and vardenafil are not so high.

The boundary potential of the membrane is composed of two components: dipole and surface membrane potentials [37,38,39]. The surface potential of the membrane depends on the presence of charged molecules on the membrane surface, while the dipole potential is determined by the orientation of dipoles of lipid head groups and water molecules in the interface. Taking into account that most molecules of PDE-5 inhibitors under these conditions are electrically neutral (Table 1), one can suppose that the ability of these agents to modify membrane electrostatics might be caused by an alteration in the membrane dipole potential. The dipole moments of sildenafil and tadalafil are equal to about 6 and 2 D, respectively. The ability of PDE-5 inhibitors to modify the membrane dipole potential was estimated using a dipole-sensitive fluorescence probe, di-8-ANEPPS [40]. Figure 1c presents the dependences of the decrease in dipole potential of POPC bilayers (*φ_d_*) upon the adsorption of the tested PDE-5 inhibitors. The maximum decrease in *φ_d_*, Δ*φ_d_*(max), in the presence of sildenafil, vardenafil, and tadalafil is presented in Table 2; it is about 90, 80, and 80 mV, respectively. Comparing Δ*φ_b_*(max) and Δ*φ_d_*(max) values, one can conclude that the dipole potential makes the main contribution to the potential drop at the interface upon the adsorption of PDE-5 inhibitors. We also compared the alteration in the boundary potential of neutral POPC (Table 2) and negatively charged POPC/POPG membranes induced by PDE-5 inhibitors. Sildenafil, vardenafil, and tadalafil decrease the Δ*φ_b_*(max) of POPC/POPG membranes by 73 ± 5, 62 ± 14, and 70 ± 5 mV, respectively. In confirmation of the key role of the membrane dipole potential in the drop in the electrostatic potential at the interface during the adsorption of PDE-5 inhibitors, their effect does not depend on the membrane charge. In addition to the orientation of the dipole moment vectors of PDE-5 inhibitors in the bilayer, the depth of the immersion of the compounds into the membrane might be of significant importance [41,42,43,44].

According to Cseh et al. [30], describing the adsorption of dipole-modifying molecules, one should take into account the change in the membrane packing density of dipoles. The incorporation of molecules should depend on the rigidity of the membrane. Cholesterol is known to play a key role in controlling membrane fluidity, and its addition into the bilayer leads to the increased ordering of lipid hydrocarbon chains and a reduction in the area per molecule [45,46]. Table 2 demonstrates that Δ*φ_b_*(max) values of Chol-free and Chol-enriched lipid bilayers in the presence of PDE-5 inhibitors are practically similar, while *K* constants are characterized by 1.5–3 times higher values. These data may indicate a lower affinity of PDE-5 inhibitors to the cholesterol-containing lipid phase.

**Figure 2:**
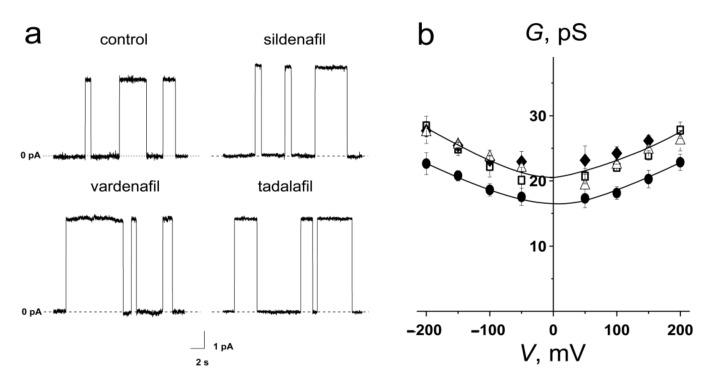
(**a**) Current fluctuations corresponding to openings and closures of single GrA channels in the absence (control) and presence of 100 μM of sildenafil, vardenafil, and tadalafil (*V* = 200 mV). (**b**) *G–V* curves of single GrA in the absence (●) and presence of 100 µM of sildenafil (□), vardenafil (Δ), and tadalafil (♦). The membranes were composed of POPC and bathed in 2.0 M KCl (pH 7.4).

To demonstrate the potential consequences of PDE-5 inhibitor-induced alteration in membrane electrostatics for reconstituted proteins, especially for ion channels triggering cellular signaling, the voltage-sensitive channels formed by the antimicrobial peptide gramicidin A (GrA) in model lipid membranes were studied [47,48]. In the case of the reduction in dipole potential, the conductance of single GrA might increase due to a reduction in the electrostatic barrier at the center of the pore for cations [49,50]. Examples of current fluctuations corresponding to openings and closures of single GrA channels in POPC bilayers in the absence (control) and presence of 100 µM of PDE-5 inhibitors at an applied voltage of 200 mV are presented in Figure 2a. The addition of sildenafil, vardenafil, or tadalafil causes practically the same increase in the conductance of the GrA channel. Figure 2b presents the *G*–*V* curves of GrA channels before and after addition of PDE-5 inhibitors. The tested compounds practically did not affect the shape of *G*–*V* dependence of peptide pores. Table 3 shows that the increment in GrA channel conductance upon introduction of PDE-5 inhibitors is about 20–25%. This value can be explained by the significant shielding of dipole potential in the aqueous pore of the GrA channel (by about 80%) [51,52]. Thus, changes in the conductance of the single GrA channels is due to a modulation of electrostatic properties of the bilayer by PDE-5 inhibitors.

### 3.2. The Effect of PDE-5 Inhibitors on the Lipid Packing and Ion Channels Sensitive to Membrane Curvature Stress

**Figure 3:**
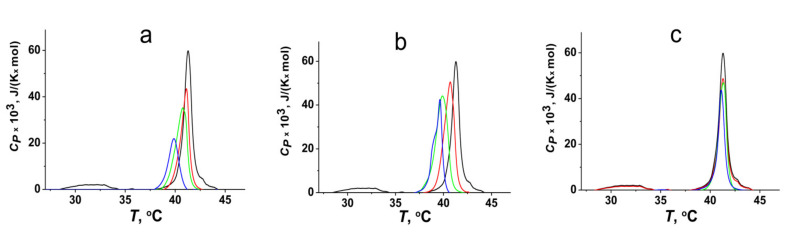
Heating thermograms of DPPC unilamellar liposomes in the absence (control, black line) and presence of sildenafil (**a**), vardenafil (**b**), and tadalafil (**c**) in the liposome suspension at concentrations of 10 μM (red lines), 50 μM (green lines), and 100 μM (blue lines).

Figure 3 illustrates the heating thermograms of the DPPC liposomes in the absence (control, black line) and presence of PDE-5 inhibitors at different concentrations. Two peaks in the absence of the tested agents are presented on the isotherms. The main phase transition is the related transformation of DPPC from an ordered gel phase to a disordered fluid state. Meanwhile, another phase transition below the main one, the pretransition, characterizes gradual elastic deformations, in which a planar membrane in the gel phase transforms into a periodically undulated bilayer, mainly due to a size mismatch between a fairly bulky headgroup of PC and its acyl chains [53,54,55]. The main transition temperature (*T_m_*) of untreated DPPC vesicles is equal to 41.2 °C, with a half-width of the peak (*T*_1/2_) of about 0.5 °C. The pretransition occurs at 34.0 °C. Figure 3a,b shows that an increase in the content of sildenafil and vardenafil from 10 to 100 µM shifts *T_m_* towards a lower temperature and significantly increases *T*_1/2_. The addition of tadalafil to the DPPC liposomes leads to a slight decrease in *T_m_* by 0.2 °C and practically does not change the *T*_1/2_ and pretransition (Figure 3c). Moreover, the adsorption of sildenafil and vardenafil on DPPC vesicles leads to suppression of the pretransition already at 10 µM (Figure 3a,b), which might indicate the strong interaction of these agents with the polar lipid head group region, while tadalafil suppresses the pretransition only at 50 and 100 µM (Figure 3c). The potent effect of sildenafil and vardenafil on the pretransition might also indicate the induction of positive spontaneous curvature stress by these inhibitors. Table 2 demonstrates the changes in the *T_m_* and *T*_1/2_ of DPPC in the absence and presence of 100 µM PDE-5 inhibitors. Changes in these parameters are able to characterize the interaction of PDE-5 inhibitors with membrane-forming lipids. The greater efficiency of sildenafil and vardenafil in affecting the lipid phase behavior compared with tadalafil may be related to a deeper insertion of their molecules into the hydrophobic region of the membrane due to the presence of the three side hydrocarbon chains, which increase the lipophilicity. Changes in the *T_m_* in the presence of sildenafil and vardenafil might be due to an increase in area per lipid molecule at the intercalation of these inhibitors into the hydrophobic membrane region.

**Figure 4:**
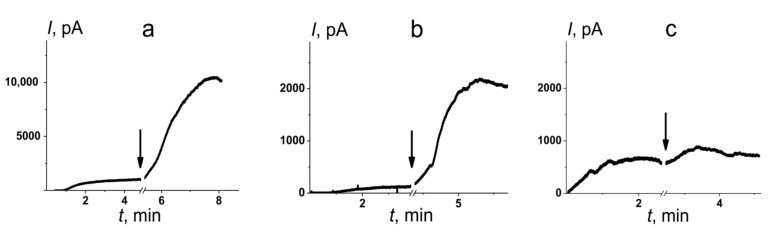
The effects of PDE-5 inhibitors on the steady-state transmembrane current induced by one-side addition of Nys. The moments of the addition of 100 µM of sildenafil (**a**), vardenafil (**b**), or tadalafil (**c**) to the bilayer bathing solution are indicated by arrows. The lipid bilayers were composed of POPC/Chol (67/33 mol%) and bathed in 2.0 M KCl, pH 7.4 (*V* = 50 mV).

To validate the possibility of an influence of PDE-5 inhibitors on mechanosensitive ion channels by altering membrane elastics, nystatin channels were tested. It is known that the single-length pores formed by the polyene macrolide antifungal antibiotic nystatin (Nys) have a lipid mouth of a positive curvature in the opposite direction to the monolayer leaflet. Low-molecular-weight membrane modifiers that induce positive curvature stress enhance the pore-forming ability of Nys [36,56]. Figure 4 demonstrates the effects of 100 µM of PDE-5 inhibitors on the steady-state multichannel activity of Nys in POPC/Chol membranes bathed in 2.0 M KCl, pH 7.4. The addition of sildenafil and vardenafil leads to a significant increase in the steady-state transmembrane current induced by one-side addition of Nys, while tadalafil slightly affects the macroscopic Nys-produced membrane conductance. Table 3 presents the mean ratios of the Nys-induced steady-state transmembrane current after and before addition of the tested PDE-5 inhibitors (*I*_∞_/*I*_∞_^0^). Sildenafil and vardenafil lead to 11- and 13-fold increases in *I*_∞_. The addition of tadalafil produces a 2-fold increase in the Nys-induced transmembrane current. Thus, the observed changes in the pore-forming activity of Nys are in agreement with the assumption about the modulation of the membrane curvature stress by PDE-5 inhibitors.

## 4. Conclusions

Summarizing the data presented, we conclude that: (i) sildenafil, vardenafil, and tadalafil affect the membrane dipole potential due to the incorporation of their dipoles into the lipid bilayer; (ii) the greater efficiency of sildenafil and vardenafil in affecting lipid phase behavior compared with tadalafil might be related to a deeper insertion of their molecules into the hydrophobic region of the membrane; (iii) sildenafil, vardenafil, and tadalafil might influence ion channels induced by the antimicrobial peptide gramicidin A via alterations in the membrane dipole potential; (iv) the changes in the pore-forming activity of nystatin are in agreement with the assumption about the modulation of the membrane curvature stress by sildenafil, vardenafil, and tadalafil.

Thus, the effects of PDE-5 inhibitors on model lipid membranes are revealed for the first time. The findings indicate that the pharmacological application of PDE-5 inhibitors requires investigation of their possible effects on the physicochemical properties of biological membranes and triggering of signaling pathways by voltage-dependent and mechanosensitive ion channels. A number of authors assume that, in addition to inhibiting PDE-5, sildenafil affects ion channels, in particular, voltage-dependent L-type Ca^2+^-channels and K_v_-channels [57,58]. Our study might expand the understanding of the molecular mechanisms of vasodilatation activity of PDE-5 inhibitors and provide a scientific and technical basis for increasing the effectiveness of treatment for erectile dysfunction and other pathologies with PDE-5 inhibitors. Characterization of the changes in the functioning of ion channels formed by antibacterial and antifungal antibiotics in the presence of PDE-5 inhibitors might contribute to the development of combined antimicrobial drugs for treatment of infectious prostatitis. Furthermore, we suppose that studying the effects of PDE-5 inhibitors on the physicochemical properties of lipid bilayers can be a precursor to the development of innovative liposomal formulations with improved pharmacological properties. However, further study of the effects of lipid vesicles modified by PDE-5 inhibitors on model and cell membranes is required. 

## Figures and Tables

**Table 1:** The physicochemical properties of the tested PDE-5 inhibitors: electrical charge (charge), the logarithm of octanol/water partition coefficient (Log*P*_o/w_), and dipole moment (µ).

PDE-5 Inhibitor	Chemical Structure	Charge *	Log*P*_o/w_	µ, D
*sildenafil*	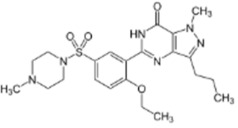	−0.34	2.7 [21] 3.18 [22]	6.4 [23]
*vardenafil*	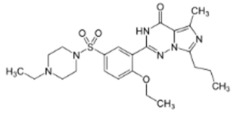	0.06	3.64 [21]	na
*tadalafil*	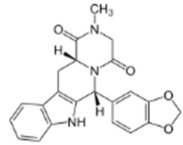	0	2.4 [24]	2.67–3.07 [25]

* The values of electrical charges of molecules of the PDE-5 inhibitors were predicted by ChemAxon; na = not available.

**Table 2:** The parameters characterizing the effects of PDE-5 inhibitors on the physicochemical properties of lipid bilayers.

	POPC			POPC/Chol		DPPC	
	−Δ*φ_b_*(max), mV	*K*, μM	−Δ*φ_d_*(max), mV	−Δ*φ_b_*(max), mV	*K*, μM	−∆*T_m_*, °C	∆*T*_1/2_, °C
*sildenafil*	67 ± 10	21 ± 4	91 ± 20	83 ± 13	30 ± 2	1.4 ± 0.4	0.7 ± 0.2
*vardenafil*	84 ± 19	17 ± 2	84 ± 23	71 ± 10	31 ± 3	0.9 ± 0.4	0.7 ± 0.3
*tadalafil*	74 ± 9	3 ± 1	78 ± 11	86 ± 10	10 ± 2	0.2 ± 0.1	0.1 ± 0.1

*φ_b_*(max) and Δ*φ_d_*(max)—the maximum changes in the membrane boundary and dipole potential, respectively; *K*—the desorption constant of the PDE-5 inhibitors; Δ*T_m_* and Δ*T*_1/2_—the changes in the DPPC main transition temperature and the half-width of the main peak at 100 μM of the PDE-5 inhibitors.

**Table 3:** The effect of the PDE-5 inhibitors on the pore-forming activity of GrA and Nys. The lipid bilayers were composed of POPC (GrA) and POPC/Chol (67/33 mol%) (Nys) and bathed in 2.0 M KCl, pH 7.4.

	*g*_sc_, pS	*I*_∞_/*I*_∞_^0^
*control*	22 ± 1	–
*sildenafil*	28 ± 1	11.1 ± 0.9
*vardenafil*	29 ± 2	12.7 ± 3.2
*tadalafil*	26 ± 2	1.9 ± 0.7

*g*_sc_—the conductance of the single GrA channels at *V* = 200 mV; *I*_∞_/*I*_∞_^0^—the ratio of the transmembrane currents induced by Nys in the presence and absence of 100 µM of PDE-5 inhibitors at *V* = 50 mV.

## Data Availability

Not applicable.
